# Case Report: Approaches for managing resistant cytomegalovirus in pediatric allogeneic hematopoietic cell transplantation recipients

**DOI:** 10.3389/fped.2024.1394006

**Published:** 2024-05-30

**Authors:** Eunkyung Song

**Affiliations:** ^1^ Department of Pediatrics, The Ohio State University, Columbus, OH, United States; ^2^ Division of Infectious Diseases & Host Defense, Nationwide Children’s Hospital, Columbus, OH, United States

**Keywords:** pediatric haplo-HCT, resistant cytomegalovirus, maribavir, viral-specific T cell therapy, CMV cell-mediated immunity

## Abstract

The instructional case is a pediatric haploidentical TCRαβ+/CD19+ depleted allogeneic hematopoietic cell transplantation recipient who developed early onset CMV infection, which was complicated by resistant CMV (both UL97 and UL54) and successfully managed with maribavir and haploidentical CMV-specific T lymphocytes. Novel approaches to resistant CMV infection are reviewed and effective utilization of recent advances in diagnosis and management of resistant CMV in pediatric HCT are highlighted.

## 1 Introduction

Cytomegalovirus (CMV) is the most common infectious complication following pediatric allogeneic hematopoietic cell transplantation (allo-HCT), occurring in roughly 30% of recipients typically between Day +30–100 post-HCT (1). CMV infection occurs from reactivation of latent infection or primary acquisition. Pretransplant CMV serostatus is the primary predictor of CMV infection, defined as CMV detection by PCR from any specimen in the absence of clinical symptoms. Although the incidence of CMV infection/disease based on CMV serostatus in pediatric allo-HCT recipients remains less elucidated, according to a large adult allo-HCT cohort (2), approximately 66% of seropositive adult recipients (R+) develop CMV infection (1), while 11% of CMV seronegative recipients of seropositive donors (D+/R−) experience primary CMV infection. Without antiviral therapy, up to 40% of CMV infection can progress to CMV end-organ disease (CMV-EOD) as defined by consensus guideline (3), leading to substantial morbidity and mortality post-HCT (4–6). Over the past decades, clinical studies have contributed to the development of advanced approaches to mitigate CMV-associated outcomes following allo-HCT. This manuscript aims to explore current and emerging strategies in the prevention, diagnosis, and management of resistant CMV infection among the pediatric HCT recipients and highlights unique challenges.

## 2 Vignette

**Figure 1:**
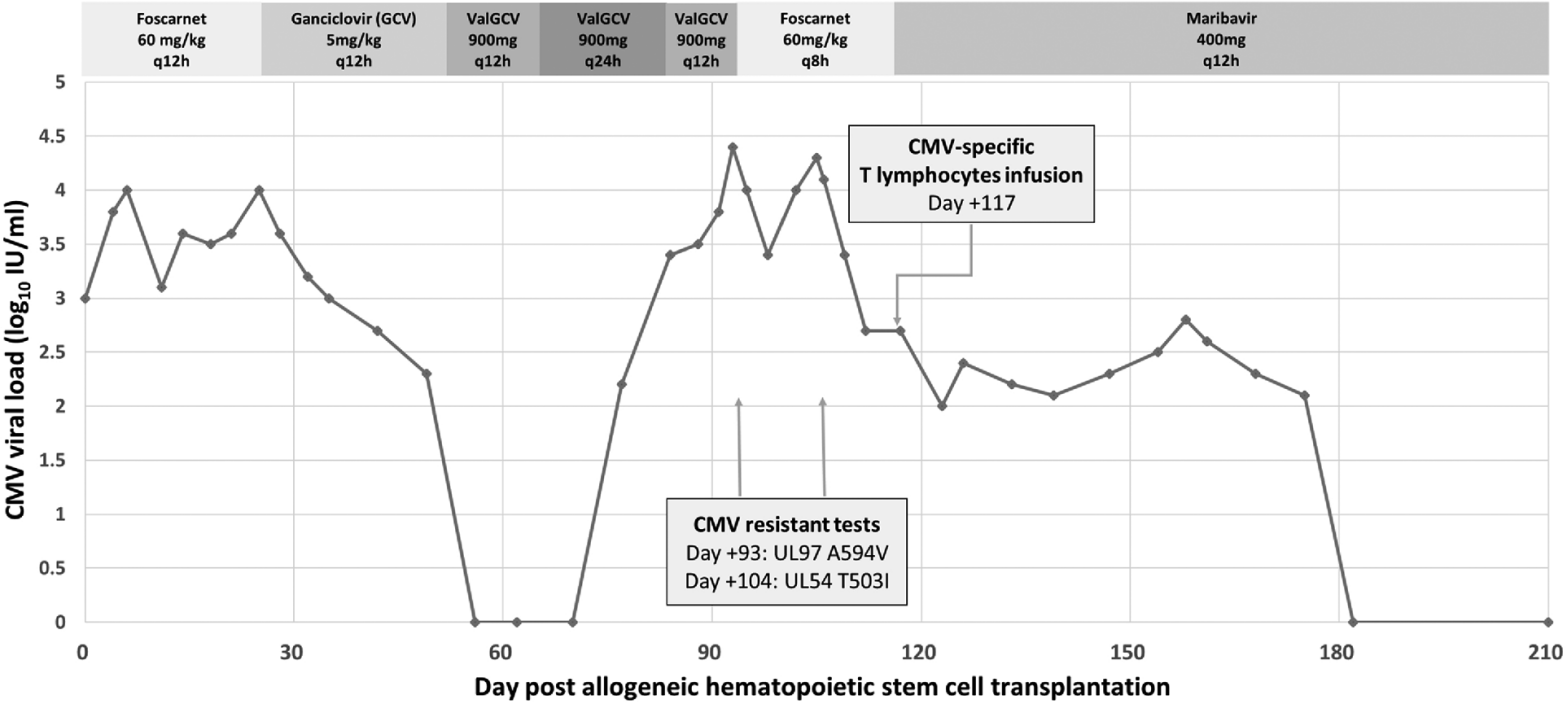
Trend of CMV viral loads and clinical events with therapy course.

A 12-year-old, CMV seropositive (R+) male with chronic myeloid leukemia (CML) underwent a maternal haploidentical TCRαβ+/CD19+ depleted allogeneic hematopoietic cell transplantation (haplo-HCT) from a CMV seropositive donor (D+). The conditioning regimen included busulfan, cyclophosphamide, anti-thymocyte globulin (ATG), thiotepa, and rituximab; no graft-vs.-host disease (GVHD) prophylaxis was provided. Weekly plasma CMV polymerase chain reaction (PCR) using a laboratory-developed quantitative PCR (7) was performed. On Day 0 post–HCT, CMV DNAemia (defined as CMV detection in plasma ≥100 IU/mL by PCR) measured at 1,052 IU/ml, without evidence of end-organ involvement (3). Pre-emptive therapy (PET) with foscarnet (FOS, 60 mg/kg/dose q 12 h intravenously) was initiated and switched to ganciclovir (GCV, 5 mg/kg/dose q 12 h intravenously) after neutrophil engraftment on Day +11 (8). Once CMV DNAemia was not detected on two occasions, 1 week apart, he was prescribed valganciclovir (VGCV, 900 mg once daily) for secondary prophylaxis starting Day +56. Recurrent CMV DNAemia occurred on Day +70, 2 weeks after starting secondary VGCV prophylaxis (Figure 1). VGCV was prescribed at induction dosing (900 mg twice daily); however, DNAemia continued to increase to 22,792 IU/ml on Day +93. Subsequently, the patient was hospitalized, and antiviral therapy was empirically switched to FOS (60 mg/kg/dose q8 h) (9) while awaiting results genotype resistance testing using next-generation sequencing (NGS, Mayo Clinic Laboratories®). At admission, the patient was clinically asymptomatic with no proven evidence for CMV-EOD, and an ophthalmology examination did not demonstrate evidence of CMV retinitis. New leukopenia (2,600/µl) and transaminitis (alanine aminotransferase/aspartate aminotransferase 123/84 U/L, retrospectively) were observed on admission. NGS genotype resistance testing ultimately confirmed the presence of a UL97 mutation at codon A594V. Of note, VGCV therapeutic drug monitoring (TDM, serum drawn at trough, 1, 3, and 5 h, Atlantic diagnostic laboratories®, PA) obtained at admission, which showed an area under the curve (AUC_0–12_) of 35 µg h/ml, below the targeted 40–60 µg h/ml for CMV treatment (10). Despite receiving 11 days of FOS, approximately a 1 log_10_ IU/ml increase in CMV DNAemia occurred on Day +104, prompting a repeat NGS test that revealed an additional UL54 mutation at T503I conferring cross resistance for GCV and cidofovir. Maribavir 400 mg twice daily orally was added on Day +107 and maternal donor-derived haploidentical CMV-specific T lymphocytes (CMV-VST) were provided as adjuvant therapy. FOS was discontinued on Day +117 after ensuring maribavir oral drug tolerance and observing improvement in DNAemia. While continuing maribavir monotherapy, DNAemia completely resolved by Day +182, with evidence of expansion of CMV specific T cells based on CMV-T cell immunity panel (Eurofins Viracor®, Lenexa KS, Table 1), presumably secondary to proliferation of CMV-VST given the absence of global T cell immune reconstitution. Maribavir was well tolerated and discontinued around 8 months post-HCT with no recurrence of resistant CMV during the 1-year follow-up post-HCT.

**Table 1:** CMV T cell immunity panel pre- and post- CMV viral specific T lymphocytes infusion.

Test (reference range)	Post HCT day		
	Day +117 (Pre CMV–VST infusion)	Day +133 (2 weeks post VST infusion)	Day +273 (5 months post VST infusion)
%CD4 CMV IFN-*γ* cells (>0.20%)	0.08%	0.33%	0.36%
%CD8 CMV IFN-γ cells (>0.20%)	0.02%	0.20%	1.04%
%CD4 SEB IFN-γ cells (>1.22%)	0.48%	0.40%	2.73%
%CD8 SEB IFN-γ cells (>1.25%)	0.02%	0.42%	3.88%
Absolute lymphocyte count	1,000 cells/ul	1,100 cells/ul	2,800 cells/ul

IFN-γ, interferon-gamma; SEB, *Staphylococcus aureus* Enterotoxin Type B.

### 2.1 Strategies to prevent CMV disease after pediatric allo-HCT: preemptive therapy (PET) vs. primary prophylaxis

Two strategies have been implemented for the prevention of CMV in allo-HCT recipients. Firstly, PET involves weekly CMV PCR monitoring during the early post-HCT period and subsequent initiation of anti-CMV drugs in patients who develop DNAemia exceeding certain viral load (VL) thresholds. However, a unified threshold has not been established and varies widely across centers (11–13). Limited adult studies examining the impact of VL threshold at the start of PET have suggested that higher CMV VLs at the initiation of PET are associated with delayed clearance of CMV DNAemia (14, 15). Nonetheless, CMV PET has effectively reduced the occurrence of CMV-EOD to <5% and prevented the progression of asymptomatic CMV infection to CMV-EOD in early post-HCT (16). This strategy has also curbed unnecessary antiviral prophylaxis in patients unlikely to experience CMV reactivation (4, 8, 17). A second strategy involves primary antiviral prophylaxis, which historically has not been an option after allo-HCT given drug toxicity. The phase 3 trial evaluating the efficacy of primary prophylaxis with letermovir, a CMV DNA terminase complex (pUL51, pUL56, and pUL89) inhibitor that blocks DNA processing and packaging and ultimately interferes with virion maturation, demonstrated a lower risk of developing clinically significant CMV infection (csCMVi, defined as CMV DNAemia or EOD leading to PET initiation) and reduced all-cause mortality in adult HCT recipients, with no apparent safety concerns (18). These data led to FDA approval of letermovir in 2017 for CMV prevention in adult allo-HCT CMV R+ recipients, and has allowed a shift to primary letermovir prophylaxis over PET for high-risk seropositive recipients (12, 18). Primary letermovir prophylaxis continues to demonstrate decrease in the frequency of csCMVi and CMV-EOD, while enhancing transplant outcomes by reducing both all cause and non-relapsed mortality (NRM) and providing better side effect profiles than GCV/VGCV or FOS in subsequent trials (19–21). In addition, letermovir prophylaxis has significantly reduced the rate of refractory/resistant CMV in adult allo-HCT recipients (22).

Letermovir is not currently approved for use in children (age < 18 years). Off-label use of letermovir as primary prophylaxis in adolescent HCT recipients has demonstrated promising efficacy (23, 24). However, limited pharmacokinetic (PK) data to inform dosing recommendations pose challenges to its use in younger, smaller children. In addition, letermovir is not universally available across all pediatric transplant centers or approved by medical insurance. A phase IIb trial assessing letermovir PK in pediatric allo-HCT has been completed, but results are pending (NCT03940586). Additionally, a phase 3 trial is ongoing to evaluate letermovir's efficacy in preventing csCMVi in pediatric allo-HCT recipients (age between 2 and 18 years and weight ≥18 kg, NCT05711667).

### 2.2 Refractory/resistant CMV: risk factors, clinical significance, and diagnosis

Resistant CMV is defined as the presence of viral genetic alterations that decrease susceptibility to one or more anti-CMV drugs while refractory CMV infection or disease is defined as CMV DNAemia increasing by >1 log_10_ IU/mL or a lack of improvement in CMV-associated signs and symptoms despite ≥2 weeks of appropriately dosed anti-CMV therapy (2).

#### 2.2.1 When to suspect resistant CMV

In this clinical vignette, genotype resistance NGS testing was obtained when the patient developed recurrent CMV DNAemia after nearly 3 months of FOS/GCV/VGCV and with a rebound of DNAemia by approximately a 1 log_10_ IU/ml while receiving FOS. The possibility of CMV resistance should be considered if: (1) CMV VL increases >1 log_10_ IU/ml despite 2 weeks of appropriate, full-dose anti-CMV therapy (refractory CMV) or after at least 6 weeks of total anti-CMV drug exposure, (2) persistent DNAemia despite proper dosing and drug adherence, (3) the development of recurrent DNAemia after prior clearance while receiving secondary prophylaxis, and (4) progression of DNAemia to CMV-EOD despite anti-CMV therapy (9). Typically, resistant CMV emerges after prolonged anti-CMV drugs exposure, often exceeding 2–3 months (9, 25). Therefore, performing CMV genotype resistance testing within the first 2 weeks of appropriate therapy is generally not warranted for anti-CMV drug naïve patients, since it is not uncommon to have modest increases in VL despite appropriate therapy during this period (25).

#### 2.2.2 Epidemiology and risk factors for refractory/resistant CMV after allo-HCT

The incidence of refractory/resistant CMV following adult allo-HCT varies. Refractory CMV occurs in 24%–39% of allo-HCT recipients (9), while resistant CMV ranges between 1%–5% in T cell-replete HCT recipients and 9%–14.5% in CD34+ selected grafts (26) or haplo-HCT recipients (27). Heightened clinical vigilance of resistant CMV is warranted, especially for recipients of T-cell depleted conditioning regimens or allografts from cord blood or haploidentical donors (9). Limited published data on pediatric allo-HCT recipients report resistance rates between 4% (28) and 10% (29). Table 2 summarizes risk factors for refractory/resistant CMV. In the clinical vignette, risk factors for resistant CMV include CMV R+ status, receipt of a haplo-HCT, prolonged DNAemia (7 weeks) until clearance, and recurrent episodes of CMV following prolonged anti-CMV drugs exposure (>2 months), likely all contributed to the development of resistance.

**Table 2:** Risk factors for refractory/resistant CMV in allogeneic hematopoietic cell transplantation.

Host factors	CMV seropositive recipient (R+) and seronegative donor (D-) Delayed immune reconstitution for CMV Improper/prolonged exposure to antiviral drugs in the presence of replicating virus
Virologic factors	Previous CMV infections with recurrent episodes of CMV Persistent or intermittent low level CMV viremia High peak level CMV DNAemia
Transplant factors	Donor type • Haploidentical donor • Mismatched or unrelated donor • Cord blood transplant Conditioning • T-cell depletion (CD34+ selection, alemtuzumab or ATG) Post-transplant factors • Acute GVHD • Post-HCT cyclophosphamide • Prednisone 1 mg/kg/day (or equivalent)

ATG, anti-thymocyte globulin; CMV, cytomegalovirus; D, donor; GVHD, graft-vs.-host disease; HCT, hematopoietic cell transplant; R, recipient.

#### 2.2.3 Clinical significance of refractory/resistant CMV

Refractory CMV may increase the risk of CMV-EOD and NRM (30). Comparison of clinical outcomes between wild-type and resistant CMV are not well-established. While not all resistant CMV episodes progress to symptomatic infection or CMV-EOD, resistant CMV significantly increases the risk of CMV-EOD and may lead to treatment failure with poor clinical outcomes, particularly in high-risk populations (2, 25). For instance, in HCT recipients receiving T cell-depleted grafts with documented CMV resistance, CMV-EOD developed in 58% of individuals. CMV antiviral drug resistance was the only independent predictor for CMV-EOD (*p *= 0.011) and resulted in a CMV-related mortality rate of 87.5% (26). Similarly, in haplo-HCT, 66% of resistant CMV progressed to CMV-EOD, with CMV-related mortality reaching 42% (27). As there is a time lapse between development of CMV DNAemia and progression to CMV-EOD, recognizing the risks associated with resistant CMV, promptly testing, and escalating empirical therapy during viral monitoring will be key to preventing progression to CMV-EOD and avoiding adverse outcomes.

#### 2.2.4 Diagnosis of resistant CMV

Confirming resistance in clinical specimens (e.g., blood, fluids, or tissues) using genotypic resistance analyses, by detecting the mutation(s) on single or multiple genetic loci that confer phenotypic resistance to anti-CMV drugs, has replaced phenotypic resistance testing given its rapid turnaround time and less labor-intensive workflow. Individual genotypic mutations are linked to various levels of antiviral resistance, as extensively elucidated in other literature (31). UL97 mutations, which confer resistance to GCV/VGCV, generally emerge first after 2–3 months of drug exposure in >90% of cases. UL54 mutations, which confer resistance to any of the traditional anti-CMV drugs with cross-resistance [between GCV-cidofovir(CDV), GCV-FOS, or triple drug resistance], may appear later. Emergent genetic mutations conferring resistant to newer anti-CMV drugs have been identified, such as UL97 mutations associated with resistance to maribavir, and UL56, UL89, or UL51 mutations associated with resistance to letemovir, as summarized in Table 3 (31, 32).

**Table 3:** Summary of anti-CMV drugs.

Drug	Mechanism of action	Indication	Excretion	Dose adjustment	Adverse effects	Genotypic resistance	HSV/VZV coverage
Ganciclovir/valganciclvoir	Inhibitor of DNA polymerase	Treatment	Renal	Yes with impaired renal function	Pancytopenia/myelosuppression (leukopenia/neutropenia), Renal injury, Diarrhea	UL97 UL54	Yes
Foscarnet		Treatment	Renal	Yes with impaired renal function	Renal injury, Electrolytes wasting, Neutropenia	UL54	Yes
Cidofovir		Treatment (salvage)	Renal	Yes with impaired renal function	Renal injury, Proteinuria, Neutropenia, Ocular toxicity (iritis, uveitis, amblyopia)	UL54	Yes if use 5 mg/kg weekly
Letermovir (PO/IV)	Inhibits viral terminase complex	Primary prophylaxis	Bile/Fecal	Not necessary with impaired renal function or mild or moderate hepatic function	Uncommon, mainly gastrointestinal (GI), dyspnea, hepatitis No significant renal and hematopoietic adverse effects	UL56 (Less commonly in UL51 or UL89)	NO
Maribavir (PO)	Directly inhibits viral kinase	Treatment of refractory/resistant CMV	Hepatic	Not necessary with impaired renal function or mild or moderate hepatic function	Mainly GI (Dysgeusia) No significant renal and hematopoietic adverse effects	UL97	NO

For the detection of genetic mutations, both Sanger sequencing and next-generation sequencing (NGS) techniques are commercially available. Sanger sequencing is suitable for targeted analysis of known mutations. However, it may not detect low-level or heterogenous populations of resistant CMV variants and requires plasma specimens with VLs ≥1,000 IU/ml for reliable analysis or for the detection of mutant strains comprising <20%–30% of the viral population to avoid false negative results (31). In contrast, NGS offers a more comprehensive and sensitive analysis, capable of detecting low-frequency mutations as well as heterogeneous subpopulations of resistant CMV during the early evolution of resistant mutation, which theoretically allows for the timely identification of resistance to prevent ineffective but toxic drug exposure (31, 33). However, NGS has not been routinely adopted in clinical practice due to its higher cost, the need for technical expertise, and challenges in interpretation requiring complex analysis and a robust bioinformatics pipeline compared to Sanger sequencing (31, 33).

### 2.3 Management of anti-CMV drugs for resistant CMV

UL97 mutations, primarily clustered at codons 460, 520 and 590–607, are associated with variable degree of GCV/VGCV resistance (9, 25, 31). Thus, clinicians may choose to continue GCV or VGCV while awaiting genotypic analysis if the risk for CMV resistance is otherwise low in asymptomatic patients. However, in high-risk patients or those with concerns for the development of EOD, an empirical change to FOS, which is independent of UL97, pending the results of genotypic resistance test should be considered. The patient in this clinical vignette had a UL97 A594V mutation, conferring high-level of resistance [a 5 to 20-fold increase in ganciclovir 50% inhibitory concentrations (EC50)] (31), required FOS induction for approximately 3 weeks. Depending on the UL97 mutation codon, the relative levels of drug resistance of GCV may vary from low- to high-level resistance; low-level resistance (i.e., <5-fold increase in ganciclovir EC 50) can be managed with higher-dose GCV (7.5–10 mg/kg q 12 h), whereas high-level resistance requires FOS (first-line option) or CDV (second-line option). Subsequent UL54 mutation may indicate cross-resistance to any of the traditional anti-CMV drugs (GCV/FOS/CDV), depending on the codons detected (9, 25, 31). In this clinical vignette, T503I resistance (cross resistant to GCV/CDV) did not alter anti-CMV management.

Following FOS induction therapy, the patient was prescribed maribavir orally as maintenance therapy. Maribavir, a benzimidazole riboside that inhibits viral UL97 kinase, received FDA approval in November 2021 for the treatment of post-transplant refractory/resistant CMV in adult and pediatric (≥12 years of age and weighing ≥35 kg) patients. This approval followed the phase 3 SOLSTICE trial, which demonstrated superior efficacy of maribavir compared to investigator-assigned therapy (IAT); maribavir achieved a viral clearance rate of 55.9% at week 8, significantly higher than the 20.8% achieved with IAT (*p* < 0.001) (34). Additionally, maribavir treatment resulted in fewer treatment discontinuations due to adverse effects compared to IAT (13% vs. 32%). Notably, neutropenia and acute kidney injury were not linked to maribavir therapy. The most commonly reported adverse event in the maribavir group was taste disturbance, experienced by 46% of participants.

However, clinicians should also be aware that virologic relapse can occur in up to 50% of subjects who initially responded to maribavir typically occurring within 4–8 weeks after discontinuation of maribavir (34, 35). Additionally, the emergence of UL97 mutations conferring resistance to maribavir, some of which confer cross-resistance to GCV, occurs not infrequently up to 25% (34–36). Therefore, monitoring CMV PCR during and after maribavir treatment is crucial, with a low threshold for genotypic resistance testing if there is an inappropriate virologic response. Maribavir's metabolism primarily relies on CYP3A4, thus close monitoring of co-administered immunosuppressant serum concentrations (e.g., cyclosporine, everolimus, sirolimus, tacrolimus) is necessary. Conversely, co-administration with drugs that induce CYP3A4 (e.g., rifampin, phenobarbital, phenytoin) leads to decreased maribavir levels and potential for reduced efficacy and should be avoided when possible. Combination therapy of maribavir with GCV/VGCV, the latter which relies on UL97 viral kinase activity, can be antagonistic and should be avoided. Maribavir has no activity against herpes simplex virus (HSV) or varicella zoster virus (VZV), thus, at-risk individuals should receive appropriate antiviral prophylaxis (Table 3). Lastly, maribavir has limited penetration into the central nervous system (CNS) and retina based on animal data, making it unsuitable for treating CMV-EOD involving the eye or CNS (35).

Maribavir is not currently FDA-approved in children <12 years of age, given lack of established safety and efficacy data. A clinical trial is currently underway to evaluate safety and PK in children and teenagers after HCT or solid organ transplantation (SOT) (NCT05319353).

### 2.4 Use of adoptive immunotherapy with CMV virus-specific T cells (CMV-VST)

In the vignette, CMV-VST were considered due to the anticipated delayed immune reconstitution following haplo-HCT in the context of resistant CMV. Donor-derived CMV-VST, generated using an interferon gamma (IFN-γ) capture technique (CliniMACS, Miltenyi®, Bergisch Gladbach, Germany) from the original haplo-HCT donor (mother), were produced per institutional protocol. CMV-VST were administered concurrently with maribavir, and we observed complete virologic clearance approximately 9 weeks post-VST infusion, with no evidence of *de novo* GVHD.

Adoptive immunotherapy using CMV-VST may be a valuable adjunctive or salvage therapy for individuals who experience drug intolerance or develop refractory/resistant CMV. Summary of recent trials in transplant recipients have been extensively reviewed in the literature (37, 38). The efficacy of CMV-VST has primarily focused on virologic response with promising outcomes, with approximately 70% of VST recipients demonstrating partial or complete virologic response. The development of GVHD post-VST infusion has been reported with rates ranging from 0 to 22%, however most cases are mild (grade I-II). Fortunately, infusional toxicities, including cytokine release syndrome, have rarely been reported (38).

However, there are significant challenges and ongoing knowledge gaps regarding VST in transplant recipients that preclude their use as adjuvant therapy as standard of care. First, accessibility to VSTs remains limited, and the development of internal VST generation requires significant infrastructure investment. An attractive option includes the use of a commercial “off-the-shelf” VST product, however accessibility currently is limited to research trials. In both cases, cost is a barrier. Additionally, determining the ideal peripheral blood mononuclear cell source (autologous vs. donor derived vs. “off-the-shelf” VSTs), method of VST generation (ex-vivo expansion vs. cytokine capture), optimal cell dose to maximize efficacy while minimizing potential alloreactivity, and establishing well-defined clinical indications and endpoints, have not been fully elucidated. Currently, multiple VST clinical trials for HCT recipients are available at clinicaltrials.gov.

### 2.5 Clinical utility of CMV immune functional assays

In the vignette, CMV-specific cell-mediated immunity (CMV-CMI) was monitored pre- and post-VST infusion using CMV-TCIP (Eurofins Viracor®, Lenexa, KS, Table 1) to assess CMV-specific immune competence. Performing functional immune assays to quantify CMV-CMI may help identify adult patients at increased risk for CMV infection and disease post-HCT in multiple studies (39, 40). However, lack of pediatric-specific data in transplant recipients limit the clinical utility of these assays in the real-world clinical setting. One commercially available assay in the United States is the CMV-TCIP. This test utilizes intracellular IFN-γ staining and flow cytometry after stimulating host T cells with CMV-specific antigens (lysates for CD4^+^ T cells and pp65 for CD8^+^ T cells) and a non-specific mitogen (*Staphylococcus aureus* Enterotoxin Type B, SEB) to determine the frequencies of CMV-specific T cells and global T cells as percentages of CD4^+^ and CD8^+^ T cells among peripheral blood mononucleated cells. Despite its availability, there are no published data to evaluate its clinical utility among at risk allo-HCT recipients.

Interpretation of CMV-TCIP results and its clinical relevance in this vignette included; (1) Negative CMV-CMI at pre-CMV-VST infusion: a negative CMV-CMI result on Day +117 post-HCT was considered a surrogate for the risk to develop refractory/resistant CMV infection and potential progression to CMV-EOD; thus providing another data point to consider the use of adoptive immunotherapy, (2) Increase in CMV-specific IFN-γ+ CD4^+^ and CD8^+^ T cells by a 4- and 10-fold, respectively at 2 weeks post-CMV-VST; this suggests that the infused CMV-VST likely expanded in response to the infection, despite persistently poor generalized T cell immune reconstitution, as evidenced by T cell responses to SEB, (3) Global T cell immune recovery at 9 months post-HCT (at 5 months post-VST infusion); there was further expansion of CMV-specific IFN-γ+ CD4^+^/CD8^+^ T cells, along with evidence of global T cell immune reconstitution by this time and the patient continued to exhibit virologic suppression, even after discontinuing secondary anti-CMV prophylaxis.

Applying CMV-CMI monitoring post-HCT to guide patient-specific therapy may allow individualize management and optimize clinical outcomes by selecting those who may benefit from adoptive T cell therapy and potentially reduce antiviral exposure and associated drug toxicities in at-risk patients. Nonetheless, further studies are needed to guide this approach in the allo-HCT population, particularly in children. Limited data have been published evaluating the clinical utility of this commercially available immune assay at this time (41). We eagerly await the results of a clinical study currently underway in pediatric SOT recipients (NCT03924219).

### 2.6 Role of valganciclovir (VGCV) therapeutic drug monitoring (TDM)

In this clinical vignette, VGCV TDM was performed to assess medication adherence and ensure adequate drug absorption in the context of resistant CMV, revealing an AUC_0–12_ _h_ of 35 µg h/ml, below the targeted range of 40–60 µg h/ml suggested for CMV treatment (10). While VGCV TDM confirms drug intake and absorption, its direct correlation with virologic or clinical outcomes remains uncertain. Wide intra- and inter-individual variability is observed, and conflicting conclusions exist regarding the relationship between drug exposure and treatment failure, development of resistance, or adverse effects (42). Given these challenges, routine (V)GCV TDM is currently not recommended. Further research is needed to evaluate the potential utility of TDM, especially in pediatric transplant recipients.

## 3 Summary

The clinical vignette illustrates the successful management of a challenging case of resistant CMV in a pediatric haplo-HCT recipient, leveraging recent advancements in diagnosis and treatment. Early identification of risk factors, coupled with the integration of novel pharmacological agents and adoptive immunotherapy, may contribute to improving overall outcomes for transplant recipients with resistant CMV infection. Furthermore, the summary highlights the need for pediatric-specific research to address ongoing significant knowledge gaps and improve outcomes in managing CMV infections in children after transplantation.

## Data Availability

The original contributions presented in the study are included in the article/Supplementary Material, further inquiries can be directed to the corresponding author.
